# Role of cancer stem cells in the development of giant cell tumor of bone

**DOI:** 10.1186/s12935-020-01218-7

**Published:** 2020-04-25

**Authors:** Abdul Rouf War, Kai Dang, Shanfen Jiang, Zhongwei Xiao, Zhiping Miao, Tuanmin Yang, Yu Li, Airong Qian

**Affiliations:** 1 Laboratory for Bone Metabolism, Key Laboratory for Space Bioscience and Biotechnology, School of Life Sciences, Northwestern Polytechnical University, Xi’an, 710072 Shaanxi China; 2 Research Center for Special Medicine and Health Systems Engineering, School of Life Sciences, Northwestern Polytechnical University, Xi’an, 710072 Shaanxi China; 3 NPU-UAB Joint Laboratory for Bone Metabolism, School of Life Sciences, Northwestern Polytechnical University, Xi’an, 710072 Shaanxi China; 4 Department of Neurology, Shanghai Pudong Hospital, Fudan University, Shanghai, 201399 People’s Republic of China; 5 Honghui Hospital, Xi’an, Jiaotong University College of Medicine, Xi’an, Shaanxi China

**Keywords:** Giant cell tumor, Giant cell tumor of bone, Giant cell tumor stromal cells, Cancer stem cells, Mesenchymal stem cells, MicroRNAs

## Abstract

The primary bone tumor is usually observed in adolescence age group which has been shown to be part of nearly 20% of the sarcomas known today. Giant cell tumor of bone (GCTB) can be benign as well as malignant tumor which exhibits localized dynamism and is usually associated with the end point of a long bone. Giant cell tumor (GCT) involves mononuclear stromal cells which proliferate at a high rate, multinucleated giant cells and stromal cells are equally present in this type of tumor. Cancer stem cells (CSCs) have been confirmed to play a potential role in the development of GCT. Cancer stem cell-based microRNAs have been shown to contribute to a greater extent in giant cell tumor of bone. CSCs and microRNAs present in the tumors specifically are a great concern today which need in-depth knowledge as well as advanced techniques to treat the bone cancer effectively. In this review, we attempted to summarize the role played by cancer stem cells involving certain important molecules/factors such as; Mesenchymal Stem Cells (MSCs), miRNAs and signaling mechanism such as; mTOR/PI3K-AKT, towards the formation of giant cell tumor of bone, in order to get an insight regarding various effective strategies and research advancements to obtain adequate knowledge related to CSCs which may help to focus on highly effective treatment procedures for bone tumors.

## Background

### Bone tumor

**Fig. 1:**
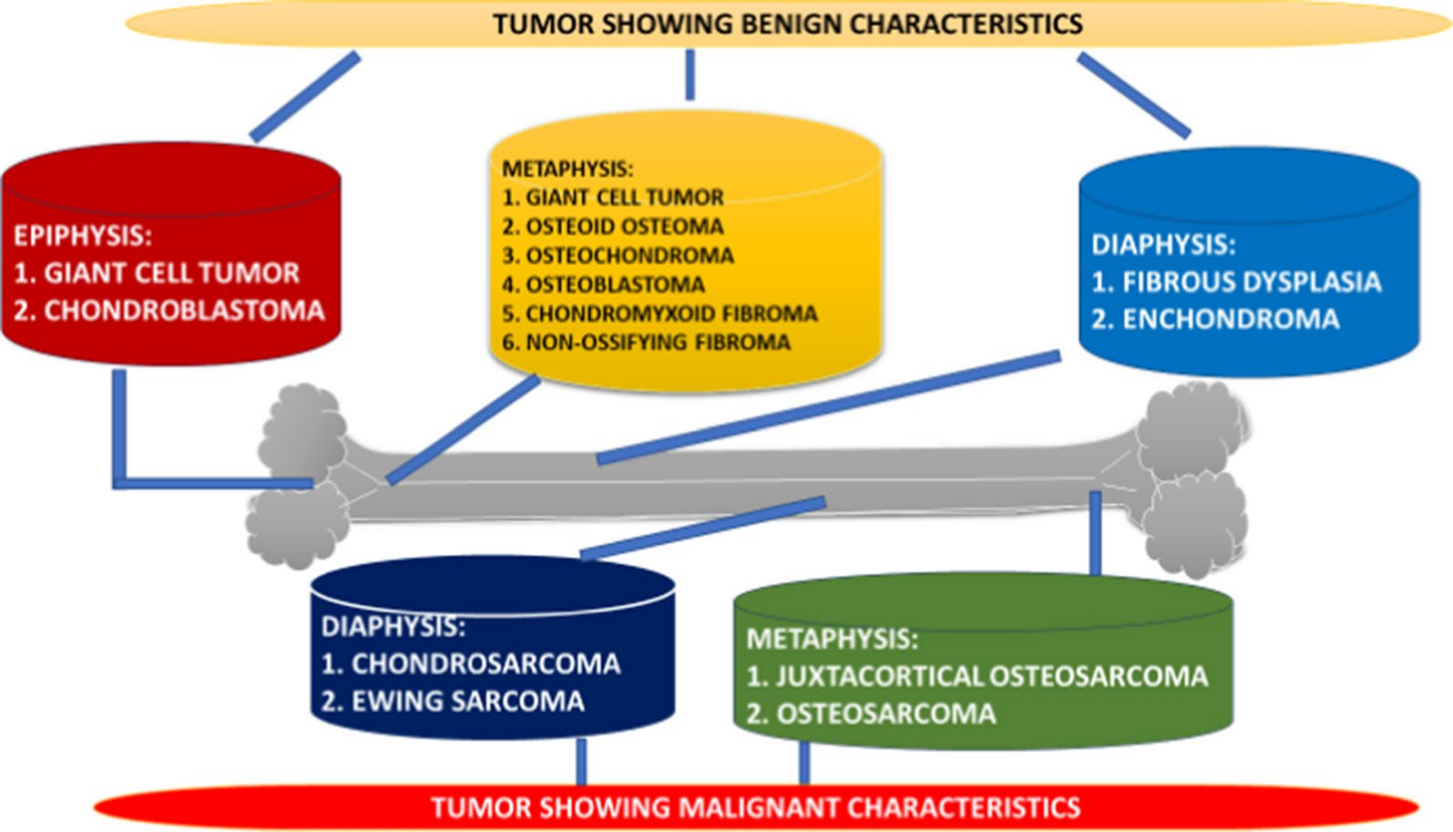
Several types of tumors categorized according to their point of origin in a bone

Bone tumor comes into existence with the help of uncontrolled division of the normal bone cells which leads to the generation of an abnormal tissue with a lump or mass of cells. Majority of bone tumors are considered to be benign (non-cancerous). Malignancy characteristics have also been confirmed in bone tumor [1]. Benign bone tumor can be normally less dangerous and mostly remain localized inside the body. In contrast, malignant bone tumors can involve other parts of the body. This process is known as metastasis [2]. Bone tumors are associated with all the bones present in the body. When a bone tumor starts developing, it targets healthy tissue and in turn makes the bone weak, which helps the bone to encounter a high risk of fracture or bone deformation [3]. A bone tumor or bone cancer can be categorized into two basic types; primary bone cancer and a secondary bone cancer. A primary bone cancer originates in a bone itself while as, a secondary bone cancer originates in the parts other than bone and with the help of metastasis reaches to the bone. Malignant tumor of bone is usually observed in adolescents and young adults. U.S. tops the list among the highest number of cases in bone tumor which estimates around four per million annually [4, 5]. Primary neoplasms of bone have been observed as non-hematopoietic malignant bone tumor which deals with the formation of osteoid matrix with the help of cells developing cancer [6]. Primary bone tumors are usually seen in young patients who are in the age group of 10–20 years. In this age group it has been found that almost 75% of these cases are related to the young adults who are less than 20 years [7]. This high percentage occurs during puberty/adolescence because there are some potential growth centers of the bone which are highly active during this period. These tumors are generally developed in the vital metaphyseal regions present in the long bones, such as; the bone present in the knee contributes around 60% of the bone tumor [8]. Secondary bone tumor is commonly observed in old age patients. Also, these tumors propagate at high level and cover much space due to prevailing context, these tumors mostly develop in flat bones [9]. Figure below (Fig. 1) shows the several tumors which occur in the different regions in a bone such as; epiphysis, metaphysis and diaphysis [10]. These several types of tumors are classified on the basis of the characteristics which they exhibit such as; benign and malignancy. These tumors occur in almost all the bones but, some of the bone tumors prefer specific locations. Bone tumor occurs at every age group but, in exceptional cases, several types of bone tumors start at certain age [11].

### Giant cells (GCs)

A tumor can be highly cellular and practically creeping or branched, it may contain some globular as well as mono-nucleated cells which include a large assembly of giant cells with complex nuclei [12]. The cytoplasm of the stromal cells is indistinct having an unspecified margin. These cells also possess a nucleus which has an unambiguous nuclear membrane and a normally bloated nucleolus [13]. The multi-nucleated giant cells exhibit similarity to osteoclasts biochemically and practically which include definite nuclei showing some closeness to the nuclei present in stromal cells. These types of giant cells are commonly seen in a number of primary bone tumors [14]. Giant cells include polyploid counterparts of chromosomes in majority, which are used for providing stability between chromosomes without leading to any sort of modification in total chromosome number. Various cancerous diseases contain more than one chromosome which is not equal in number, this inequality leads to the risk for the development of a type of deformity named as aneuploidy [15]. The high volume of the nucleus of the cell in case of giant cells results in the rapid increase of the size of various internal cellular components. The giant cell development is actually the process of either improper or absence of mitotic phase. The three typical methods which result in the generation of giant cells in a regular fashion are; (a) mitotic catastrophe, (b) cell fusion or (c) endo-replication process [16]. These methods function either individually or in a combined manner. Giant cell is usually large in size which contains multiple nuclei. This type of cell is produced by the process of fusion of various cells such as macrophage, epithelioid cells, monocytes, etc. which work together in an integrated fashion [17]. The area of chronic inflammation and other granulomatous environment are the abode of giant cells. The phenotypic variation in multinucleated giant cell types is based on the restricted atmosphere and the physicochemical characteristics of the targets which are responded via. signaling by giant cell types and their monocyte/macrophage precursors [18]. It has been found that certain important factors for example; dendritic cell specific trans-membrane protein (DC-STAMP), macrophage fusion receptor (MFR), an adhesion protein, vitronectin and a fusion factor help in the production and function of giant cells in normal manner. These giant cells play a potential role in pathological aspects which makes these cells to work as specific diagnostic tools in osteology area [19]. The multinucleated giant cells which are known till date are; giant cells associated with mycobacterium-induced granulomas, several giant cell tumors of bone, production and function of osteoclasts, and giant cell generation and function of foreign body [20].

### Polyploid giant cancer cells (PGCCs)

As per the previous reports, human solid tumors possess several different copies of DNA in a large number of cancer cells which are anomalous and contribute effectively to their histopathologic characteristics. These types of cancer cells are called as polyploid giant cancer cells (PGCCs) [21]. In case of post-chemotherapy, severe morbid conditions and post-malignancy, PGCCs have been observed to exhibit high variation and distinction in terms of size. These type of cancer cells at nuclear level play a vital role in the diagnosis of prognosis in ovarian cancer [22]. The important process involved in the formation and development of giant cells is the mitosis/cytokinesis failure which occurs repeatedly thereby working for the genomic instability set up. PGCCs can be isolated from several cancer cell lines with the help of CoCl_2_ and these types of cells can be maintained effectively in vitro [23]. It has been investigated recently that PGCCs resemble to various simple organisms in case of cell proliferation process. PGCCs may act as a quiescent set of cancer cells which are subjected to stress in their cancer cell life cycle. This stress condition developed in the cells helps in the growth of cancer stem-like cells which plays a major role in tumor formation [24]. Recent studies show that a wide range of markers for stem cells are expressed by PGCCs, these cancer cells also give rise to daughter cells with the help of irregular division process thereby forming spheroids as well. The daughter cells produced by PGCCs under in vitro conditions were shown to be highly tumorigenic as compared to the cancer cells which are of normal size and produced in nude mice [25]. It has also been shown that these PGCCs are associated with a mesenchymal phenotype which when prompted into several benign families like; bone and cartilage, adipose tissue etc., help in the exhibition of cancer stem cell-like characteristics in PGCCs. The PGCCs which have been raised with the help of CoCl2 have been found to be much more steadfast with discrete morphology shown by these cells in the culture conditions which possess relevant characteristics [26]. A sarcoma cell line model was used by researchers recently in which it was confirmed that multinucleated giant cancer cells contribute effectively towards the development of tumorigenicity as compared to typical cancer cells. In eukaryotes, the cell division occurs by mitosis but a wide range of alterations are seen in mitotic cell cycle which under stress conditions can help in the fulfillment of the requirement of growth and development [27]. The process of end archetype or endocycle is the same alteration taking place in normal mitotic cell cycle which includes several steps in DNA replication process irrespective of mitotic course of action. With the help of this process multinucleated cells get developed. The same process plays an important role in the growth patterns observed trophoblasts, insects etc. [28]. Various factors like aging, stress and unproductive cell cycle can be very much helpful in case of raising PGCCs. The generation of PGCCs is the result of cell fusion process which is considered as an important factor for cancer progression [29]. It has been confirmed that several chemotherapeutic drugs which are used to block mitosis help in the acceleration of giant cancer cell formation, these cells are usually shown to be susceptible to the holocaust involved in mitosis along-with the later step for apoptotic process in a sequential manner [30]. PGCCs may undergo the process of budding and bursting in order to give birth to the daughter cells. The eukaryotic diploid cells in contrast undergo conventional growth pattern in mitosis which shows a visible distinction [31]. Giant cells can be subjected to reciprocation process with the help of depletion in the division, which leads to the reformation into the cancer cells having definite pattern. This entire process can be referred to as neosis or depolyploidization [32]. Neosis or reductive cell division occurring in giant cancer cells mediated by meiosis-like depolyploidization has been shown to be highly effective in understanding the new life cycle changes taking place in these cells. PGCCs possess a number of features which help them to gain potential in tumor development [33]. PGCCs experience epigenetic modification as well as DNA recombination process in several copies of genes these cells contain. These steps add to the capability of PGCCs in order to develop several cell types with a wide range of cellular functions. The variation in metabolism set up is shown in PGCCs as compared to uniformly arranged cancer cells. This variation in PGCCs helps them to habituate various types of microenvironments related to hypoxia and stress conditions. The processes like budding, bursting helps the PGCCs to produce a wide lineage of cells. These processes play an important role in the process of removal of viruses in the host cell which is contaminated [34]. The morphology of PGCCs is said to be similar to the spores present in lower organisms. Recent research studies have confirmed that the cancer cell life cycle of PGCCs is experimental in which stress conditions are propagated being conjured with the help of archaic, highly controlled and metamorphic in execution which is taken into account by the cancer cells in order to deal with stress and also to fulfill the requirements to help in rapid reproduction. These characteristics exhibited in PGCCs allow their recognition and huge contribution in cancer cell life cycle as well as in the development of diversification, growth and chemo resistance in the tumor [35].

## Giant cell tumor of bone (GCTB)

**Fig. 2:**
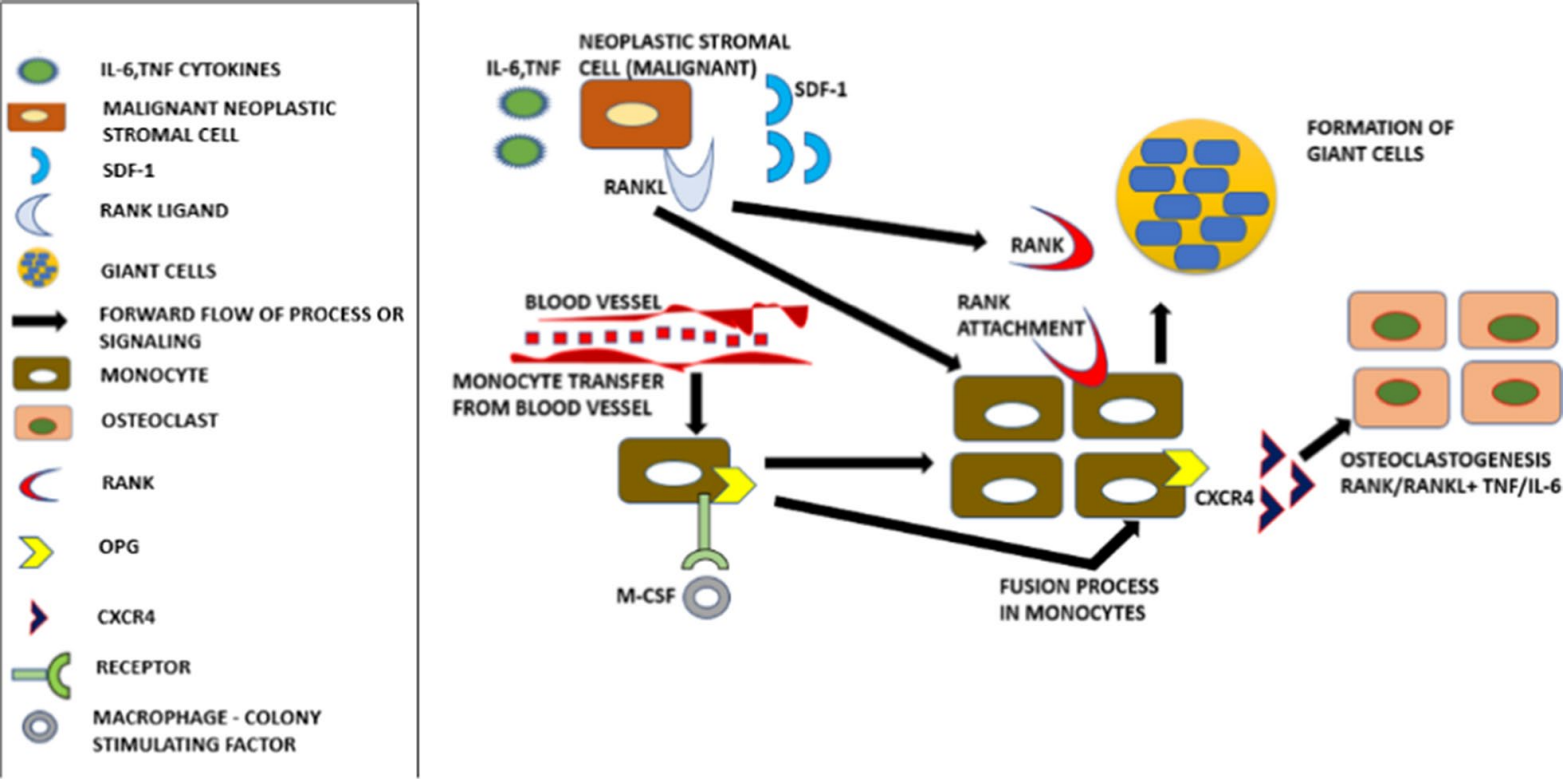
Giant cell formation by the process of fusion of monocytes through RANK-RANKL signaling system along-with several factors such as; SDF-1, M-CSF, IL-6, TNF etc. which give rise to the production of osteoclasts

Giant cell tumor of bone (GCTB) consists of three distinct cellular constituents. (1) Circular shaped mononuclear cells, (2) Spindle-like stromal cells, and (3) Osteoclast-like multinucleated giant cells [36]. Stromal cells contribute to the involvement of tumor-related myeloid lineage cells and also help in the establishment of osteoclast-like giant cells which lead to the bone desorption processes. Several features of stromal cells with the help of their unique characteristics play a critical role in the development of neoplasm in GCTB [37]. These cells under both in vitro as well as in vivo environment have undergone propagation at an increased rate which have been proliferated in a monolayer cell culture to several sub cultures and in immune-compromised mouse, these cells developed tumor post-implantation process [38]. GCTB has been found to be significantly voracious neoplasm which currently has the presence equal to approximately 5% of all the primary bone tumors at global level. This tumor includes various factors such as; specific localization, malignancy and modifications related to metabolism which helps this type of bone tumor to influence the radiographic and histopathologic findings [39]. Young adults are prone to conventional/primary bone tumor at major level. The revelation of malignancy in case of giant cell tumor has been shown in the form of sarcoma developing in giant cell tumor. A primary malignant giant cell tumor is the sarcoma of giant cells whereas the tumor present on the pre-condition which is detected in giant cells is named secondary malignant giant cell tumor [40, 41]. The malignant fibrous histiocytoma or fibrosarcoma are produced in primary giant cell tumor and these sarcomas are also produced in secondary malignant giant cell tumor. A distinctive type of secondary malignant giant cell tumor designates carcinosarcoma as a malignant mass being developed in giant cell tumor [42]. The giant cell tumor of bone can develop in meta-epiphyseal region and emerge after the completion of maturation process in the skeleton. When the tumor generation begins at the origin, mono-nuclear histiocytic cells help in the integration of the tumor due to which the process of fusion is started and the formation of multi-nucleated giant cells (MGCs) tumor takes place [43]. The receptor activator in nuclear factor kappa B ligand (RANKL) expression occurs due to the stromal cells of neoplastic tumor included in giant cells which helps in its combination with an important cofactor, macrophage colony stimulating factor (M-CSF) [44, 45]. Monocytes are helped by stromal cells in the provocation of multinucleated giant cells (osteoclastomas) production. Various macrophage markers are expressed by CD68-positive monocytes with the help of MCP-1, SDF-1 etc. produced by stromal cells. Previous reports confirm that the production of VEGF takes place through the stromal section. VEGF combines with CD68 cells to help them in expressing VEGFR1 (Flt1). GCT stromal cells producing VEGF can be hypervascular tumor in which intratumoral bleeding is possible. RANKL molecule plays a potential role in GCTB which helps the researchers in the deep observation of the pathophysiology of this disease [46]. Murine thymoma line was used initially to identify RANKL in order to explain TNFR homologues which helped the researchers to find its role in the formation of osteoclasts. Immature osteoblast-like stromal cells in GCT secrete RANKL which helps in engaging monocyte precursors to develop osteoclast-like giant cells in tumor [47]. In this entire process, RANK is expressed by activation of monocyte precursors with the help of Macrophage-colony-stimulating factor (M-CSF) which is released by the stromal section. Monocytes which are engaged in this process also experience differentiation, proliferation events along with M-CSF [48]. GCTB cases have shown significant increase in RANKL expression when compared to control samples. Monocytes which are engaged in the development of GCTB undergo cellular fusion incited by RANKL which result in the development of multinucleated giant cells characteristics of this disease (Fig. 2). The infected stromal section comes in contact with these giant cells through paracrine signaling which introduces the development of multinucleate giant cells. The contribution of soluble RANKL is also possible in this case. The serum of patients with GCTB exhibited increased levels of RANKL as per the reports [49]. The giant cells formed in GCTB act like osteoclasts in physiological manner. These cells show the expression of cathepsin K and vacuolar H^+^ ATPase which are recruited for the degradation of the hydroxyapatite and organic constituents of bone, causing the osteolytic lesion of the bone. Also, GCTB shows increased levels of a variety of matrix metalloproteinase which play important role in bone degradation as well as stromal reformation [45].

Giant cell tumor is basically composed of several types of cells such as; mononuclear histiocytic cells, multi-nucleated giant cells and these cells work in combination under a specific environment called as; monocytic-histiocytic system [50]. Giant cell tumor stromal cells (GCTSCs) are the cells which belong to neoplastic tumor and exhibit proliferation at high level. Nishimura et al. in 2005 determined the process of introducing the multinucleated giant cell production with the help of some important soluble factors from the monocytes in GCTSCs [51]. Distinctive biomarkers associated with osteoclasts are observed in multinucleated giant cells. The gene expression in GCTSCs helps in the identification of the osteoblastic differentiation at initial stage in case of stromal cells and some important characteristics of mesenchymal stem cells have also been observed [19]. Giant cell tumor (GCT) undergoes the lysis process in the middle portion present in the outer region, it also involves the metaphyseal point particularly and elongates to the connecting articular cortex [52]. It has been observed that less than 2% of the area where the lysis occurs, is existing in the metaphysis or diaphysis and also most of the changes take place in the intramedullary region in the long bones including femur and tibia. These abrasions show prominent regularity and have been shown to be positioned at central location while growing unconventionally [53]. Giant cell tumor of bone (GCTB) being a primary bone tumor exhibit several significant biotic features which are composed of some important types of cells which are significantly different at histological level, these features are observed in the cells which include; Osteoclast like multi-nucleated giant cells, the spindle shaped cells, fibroblast-like mesenchymal stromal cell, and a discoid morphology called macrophage-like cells [54]. Table 1 gives an overview about the role played by polyploidy giant cancer cells, cancer stem cells, miRNAs in the development of normal bone tumor as well as giant cell bone tumor [55]. World Health Organization has grouped GCT into a benign tumor which in contrast exhibits extremely critical indigenous antagonism, this tumor also shows the susceptibility towards native recurrence particularly in spine along-with the process of metastasis [56]. In case of initial level treatment methods or impulsive malignant transformation regardless radiation therapy, malignancy changes such as; sarcomatous transformation is observed in GCTB. Cooper in 1818 demonstrated this type of tumor and with the passage of time, GCTB has gained much more popularity which helped in the recent research studies which show the significant role played by prognostic parameters in the development of GCTB [57].

**Table 1:** Differentiating between normal cells of bone and giant cell tumor of bone on the basis of several factors responsible for their development

Bone cells/ giant cell bone tumor	Cancer stem cells (CSCs)	mTOR/PI3K-AKT Signaling (CSC-Based)	Mesenchymal stem cells (MSCs)	MiRNAs gene expression (CSC-Based)	Mutations	References
Normal cells of bone	(i) CSCs are not present (ii) Stem cells are present as small population. (iii) CSCs play no role in skeletal development in bone cells	(i) Plays regulatory role but no CSC-based signaling present. (ii) Participates in bone cell metabolism (iii) Plays role in cell proliferation, cell cycle progression and survival	(i) Multipotent in nature. (ii) Present in various adult tissues as well as bone (iii) MSC markers express through sub-population of stromal cells	(i) Small noncoding, single-stranded RNA molecules present (ii) miRNAs are important part of normal bone cells. (iii) Normal gene expression in bone cells	No mutation occurs in normal bone cells	[70, 72] [99, 102], [125, 129], [64, 67],
Giant cell tumor of bone (GCTB)	(i) Over-expression occurs. (ii) Undifferentiated in nature (iii) Play role in tumor initiation, proliferation and its maintenance	(i) Overexpression occurs in GCTB. (ii) Helps in the conversion of CSCs to endothelial cells (iii) Helps CSCs to maintain stem cell characteristics like; self -renewal, survival and cell proliferation	(i) Participates in activation and proliferation of giant cells in Bone tumor. (ii)MSCs/GCTSCs differentiate into osteoblasts, adipocytes and chondrocytes (iii) Expression of MSC markers confirm the development of GCTSCs	(i) Mutations at insubstantial locations in the chromosomes where gene coding occurs for miRNAs in CSCs (ii)Mediate giant cells and osteoclasts by regulating osteoclastogenesis. (iii) Act as potential biomarkers to detect several types of cancers	Mutations occur in Giant cells, stem cells, normal cells of bone	[75, 78] [111, 114], [126, 129], [141, 142]

It has been reported that approximately 15 to 20% cases of non-malignant bone lesions are present in Giant cell tumors (GCTs) which are usually recognized as stage 2 or 3 lesions. These tumors are benign in nature as well as they sometimes show localized intrusiveness and nearly 50% of these tumors undergo a characteristic recurrence process post-curettage [58]. A number of treatment methods have been designed so far in order to treat this tumor, these treatment methods are; chemical, thermal, mechanical, biotic, injection etc. These treatment methods were suggested to control this dreadful disease with the help of eradicating the tumor through curettage process [59]. The giant cells play a main role in the expression of calcitonin receptors which has been reported recently, giant cells mainly consist of osteoclasts in chronic giant cell granulomas (CGCGs). This fact was confirmed with the help of immune-histochemical study in which osteoclast specific monoclonal antibodies were used [60]. GCTBs undergo secondary modifications including aneurysmal bone cyst (ABC) modification, foamy histiocytic combinations and responsive bone or osteoid production [61]. It has been found that several types of bone lacerations involving osteoclastic giant cells such as; osteosarcoma, primary ABC, non-ossifying fibroma, chondroblastoma, brown tumor (hyperparathyroidism), giant cell reparative granuloma and the giant cell-rich type specifically exhibit much more distinction than GCTB. Recurrence in case of tumor post-treatment usually takes place after 3 years but, GCTs have shown late relapse such as 15 years of time span which occurs at the locus where surgical treatment was performed to eliminate the tumor [62, 63].

## MicroRNAs in GCTB

MicroRNAs are classified as small noncoding, single-stranded RNA molecules that trigger degradation or inhibit the translation process in mRNA molecule which results in the de-regulation of their target genes. Moderate or comprehensive binding of miRNAs to the 3′-untranslated region (3′-UTR) of their target mRNAs actually represents their function in which these molecules block the translation or degrade the mRNA [64]. MicroRNAs were initially revealed by Lee et al. 1993 in *Caenorhabditis elegans*. Biogenesis mechanism in miRNAs usually operates from transcription of intergenic, intronic or polycistronic loci by RNA polymerase II as observed by Lee et al., in 2004 and Borchert et al. in 2006 to an 80-nts, capped, polyadenylated pri-miRNA transcripts which are associated with a stem-loop unit [65]. According to Bioinformatics study, miRNAs are capable of regulating nearly one-third of entire mammalian gene setup. Abnormal expression of miRNAs creates the pathological conditions leading to cancer. There are less reports showing the function of miRNA-induced osteolysis in GCT. The development of giant cells in GCTB and osteoclasts is mediated by miRNAs by regulating osteoclastogenesis [64]. Recent reports show that miRNAs are usually needed in the process of osteoclastogenesis, which helps the researchers to understand the blockage of V-maf musculoaponeurotic fibrosarcoma oncogene homolog B (MAFB) and RANK expression by ectopic expression of miR-148a and miR-503, which in turn leads to the inhibition of osteoclast differentiation. The role of miR-126-5p in osteoclastogenesis mediated by MMP13 and PTHrP molecules as potential targets in GCT has been confirmed recently [66]. Noncoding microRNAs (miRNAs) have been shown to act as important elements to be regulated in vast majority of tumors. miRNAs have been confirmed as important biomarker in the determination of several types of cancers such as; gastric cancer. miR-16-5p expresses in breast cancer [67]. Shang Sang et al. in 2017 investigated the underlying function of miR-16-5p in GCT, in which its expression was detected in patients with GCTB. Osteoclast formation was shown to be increased, and a marked decrease in miR-16-5p expression along with significant bone destruction was shown. This outcome claimed that miR-16-5p might be a potential target in the pathogenesis of GCT [68]. It was also reported that there was considerable decrease in miR-16-5p levels in vitro in RANKL-induced osteoclastogenesis progression taking place in BMMs. miR-16-5p was also shown to decline RANKL-induced osteoclast development. Collectively, these results proclaim that miR-16-5p blocks the process of osteoclastogenesis and this miRNA may act as a therapeutic target in case of giant cell tumor of bone [68–70].

## Role of several important factors in the development of giant cell tumor of Bone (GCTB)

### Role of cancer stem cells (CSCs) in GCTB

**Fig. 3:**
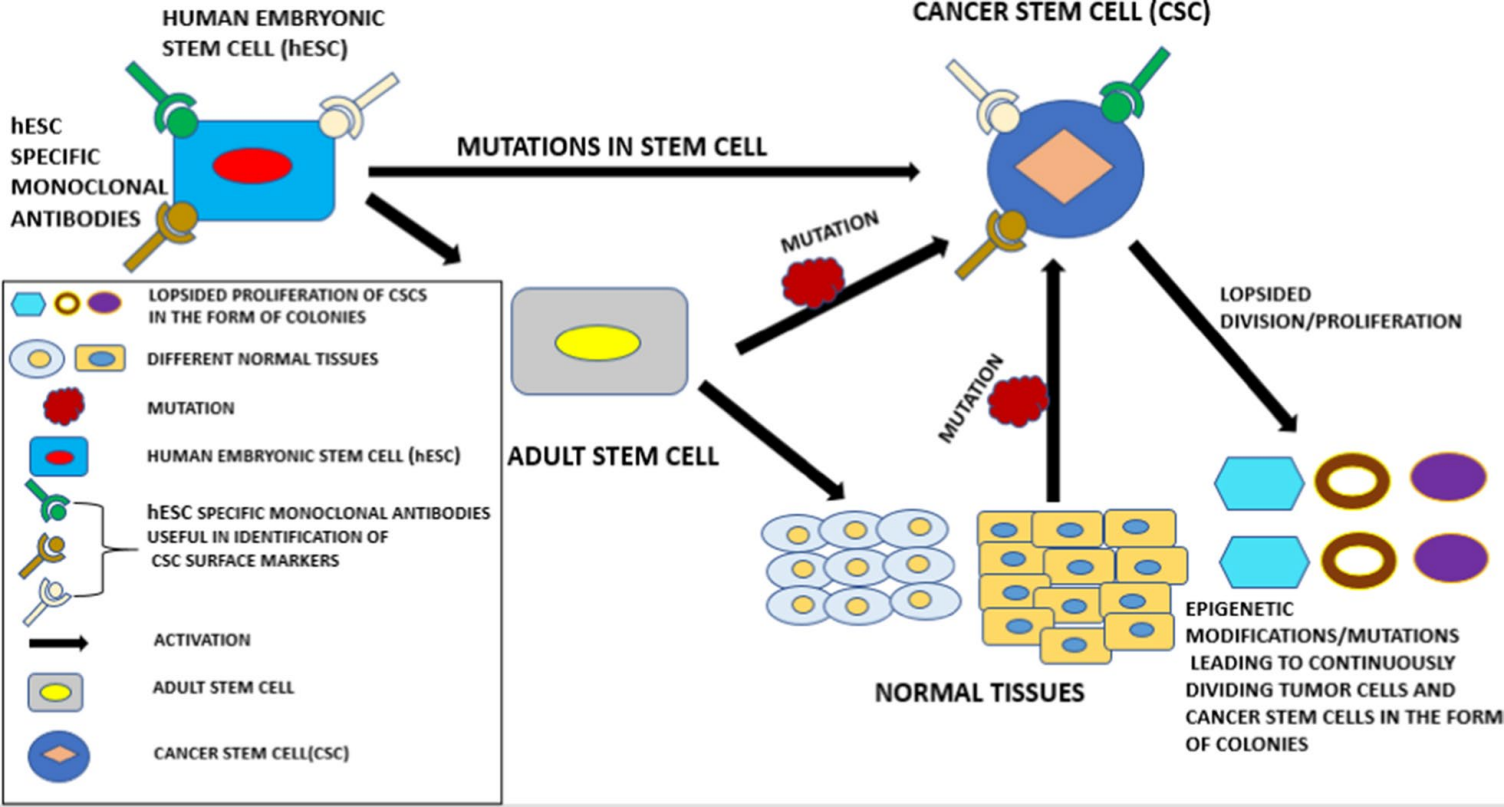
Human embryonic stem cells (hESCs) and other normal stem cells like; adult stem cells shown in the figure may lead to the development of normal stem cell progeny as well as other tissues normally, but when mutated, it leads to uncontrollable hierarchy giving birth to enormous cell types with mutations forming huge mass/colonies at metastasis stage considered as cancer stem cells (CSCs). Monoclonal antibodies (MAbs) could be present in cancer stem cells at high level which can be highly specific to embryonic stem cells and play a marvelous role in the identification of suitable surface biomarkers for cancer stem cells

Cancer stem cells(CSCs) being considered as a sub-population of the cells susceptible to tumor generation which are undifferentiated, have been identified to play a vital role in the process of tumor initiation, proliferation as well as its maintenance [71]. These cells possess the capability of high level of proliferation potential, self-renewal and the ability of developing a niche of differentiated cells including tumor population in majority [72]. Taking cancer stem cell-based model into consideration, normal stem cells act as proto-tumorigenic cells which consist of important features related to the cells which exhibit malignancy as well as indigenous start-up of important pathways essential for survival and the potential of horizonless proliferation (Fig. 3) [73]. All types of stem cells such as; pluripotent stem cells, totipotent stem cells etc., when suffering from unusual mutations result in the development of cancer stem cells. In the figure below, for example; normal human embryonic stem cells (hESCs) bound by specific monoclonal antibodies, undergo mutations which lead to cancer stem cells and evade apoptosis (programmed cell death). These cancer stem cells are present inside the tumor mass which help in the proliferation, invasion and other processes in cancer. The boundless growth/increase of cancer cells occurs due to the oncogenic mutations taking place under suitable conditions in which the properly modulated growth strength in stem cells under normal environment convert into peculiar cancer cells at a high rate [74]. Hypoxia has been shown to contribute in the development of tumor and specifically plays an important role in the production as well as alimentation of cancer stem cells. It also helps in the endorsement of stem like phenotype and tumorigenicity characteristics in the tumor [75].

Cancer stem-like cells have been identified and characterized in various solid tumors such as; melanoma, breast, brain, prostate, pancreatic, colon carcinomas as well as bone tumors/giant cell tumors as suggested by the recent reports [76]. The basic pattern of the development of a cancer stem cell in a tumor has been depicted in Fig. 3 above, in which human embryonic stem cells lead to the generation of CSCs via. several routes. These cells can also be maintained under in vitro conditions in the form of tumor cell colonies [77]. Along-with this process, the original tumor can be developed into immune-deficient mice after successful transplantation. Recently, several markers related to cancer stem cells have been derived which play an important role in stem cell research. Both hESCs and CSCs can possess the similar cell surface markers. Specific monoclonal antibodies(mAbs) present in hESCs could be highly useful in the discovery of various CSCs surface markers. These specific mAbs may or may not be present in normal tissues or adult stem cells developed by hESCs via. signaling mechanism. It has been reported that hESCs and CSCs resemble to each other in several characteristics such as; multipotency or pluripotency, unspecified self-renewal, elevated proliferation tendency, overexpression of anti-apoptotic genes, increased nuclear to cytoplasmic fraction, and these cells could express identical antigens which are oncofetal in nature, for example; TRA-1-81, SSEA-4, Cripto, EpCAM etc. When ESC-like gene expression in adult cells gets activated, it provides an opportunity for CSCs to exhibit self-renewal characteristics. Among all the CSC surface markers identified so far, most of these markers are expressed on adult stem cells or normal embryonic stem cells [78]. It has also been found that brain, hematopoietic, prostate and colon cancer stem cells possess the antigen CD133 observed in the membrane, which exhibits the same level of expression as shown by normal stem cells in various lineage patterns [79].

GCTB being considered as benign tumor as well as aggressive at local platform has been confirmed to exhibit local relapse [80]. The various forms of therapies prevailing today show some potential failure in the treatment of GCTB and the reason behind this failure has been confirmed to be the accumulation of stem cells which essentially contribute to the several characteristics such as; tumor initiation and relapse [81]. Tumor Stem Cells (TSCs) have been observed in a wide variety of tumors, and contribute effectively towards the startup process in the tumor development in order to help in the continuous growth of the tumor. TSCs exhibit several characteristics like formation of spheres, multipotency, rapid growth and multidrug resistance as well as the expression of Stro-1 antigen. In this regard, it was conferred that TSCs must be stro-1 positive cells in case of GCTB. TSCs present in GCTB show a pivotal role in multipotency with the help of which these cells differentiate into osteogenic and adipogenic patterns [82]. The carry-on procedure related to the osteogenic differentiation tendency is observed in stro-1 cells which are assumed to be associated with the osteoblastic origin of stem cells [83]. Recent reports confirmed through IF staining processes that pre-osteoblast protein molecules such as ALP, COL1 were developed by Stro-1 small population. The Stro-1 co-expression with ALP in cells in the middle of the process of osteogenic differentiation was confirmed by Gronthos et al. [84, 85]. In this case, it was demonstrated that osteoblastic progenitors after complete transformation lead to the development of TSCs in stro-1 cells in GCTB which helps them to obtain multipotent nature. The stem cells also exhibit differentiation capability and give rise to osteoblastic lineage [86]. Expression of some important stem cell related genes such as OCT3/4, SOX2, NANOG etc., helps in the overall growth and development of TSCs in GCTB, which in turn helps in the self-renewal and nuclear reprogramming processes in order to maintain their functional characteristics [87]. Asymmetric cell division plays a vital role in the development of self-renewal, proliferation properties in the cancer stem cells which help Tumor stem cell to go through division process in order to develop into one identical stem cell and one progenitor tumor cell. After this process the later cells propagate at a high rate which leads to the differentiation process of these cells into non-tumorigenic tumor cells. In case of suspension cultures, the propagation rate gets subsided for TSCs in GCTB, this process is predominantly beneficial for self-renewal potential in case of adherent cultures [88–90].

The histological appearance of an increased amount of osteoclast-like giant cells according to Jaffe et al. in 1940 was considered to be the neoplasm of the osteoclast lineage and hence it was given the name “osteoclastoma”. With the help of further studies at later stage, it was observed that the neoplastic component was the fibroblast like stromal cell [91]. Recent research studies have confirmed that the osteoblast lineage could consist of stromal cells along-with other sub-cell types. Under in vivo conditions, the generation of mineralized nodules by GCT stromal cells (GCTSCs) was observed in case of transplantation inside immune-deficient SCID mice. Also, in case of in vitro process, an osteoblastic differentiation of GCTSCs was found to occur [92]. According to previous reports, the multi-nucleated GCT cell line, GCT23 was described to be the cell line with potential use in GCT research. The three cellular components, multinucleated giant cells, CD68-positive monocytes, and mono-nucleated fibroblast-like stromal cells, when cultured under controlled environment showed that only the stromal cells have the ability to remain consistent with adequate percentage of viability survival making, thus these cells are considered to be highly reliable for GCT study. This might happen due to few reasons that these components which show negligible viability may not be neoplastic components, stromal cells could be neoplastic in nature which helps them to survive [93]. GCTSCs being the only potential cell type in cell culture have been confirmed to undergo cytogenetic aberrations including telomeric fusion, aneuploidy, and chromosome deletion. This was recently confirmed with the help of combined approaches that MSC population helps in the development of GCTSCs. In this case, the antigen expression pattern of the GCTSCs (which were investigated by researchers recently) and commercial MSCs used in the study completely matched when compared in various aspects [94]. It was also found that nearly all GCTSCs known so far, exhibited positive staining for the newly characterized antibodies such as SH2, SH3, SH4, and anti-CD166. A set of frozen samples of GCTs were studied as well in which a significant amount of mononuclear cells were found to exhibit positivity as a result of staining with biomarkers such as; CD166, CD105, and CD73 antigens [95].

GCTB can be categorized into several multinucleated cell types such as; fibroblast-like stromal cells, osteoclast-like giant cells, CD68 + phagocytic histiocytes. According to the previous research it has been shown that the stromal cells act as the neoplastic cell population, which mostly originates from mesenchymal stem cells (MSCs). MSCs have been found to play a vital role in the development of several types of tumors like; bone tumor [96]. It has been hypothesized that cancer stem cells (CSCs) contribute to a greater extent in several processes involved in cancer such as; growth, invasion, metastasis and multidrug resistance [97]. The pre-active mechanism of defense and survival present in CSCs may help them to withstand the conventional therapeutic methods in case of cytotoxicity in the tumor volume. As the CSCs possess the characteristics of self-renewal and differentiation processes, these characteristics help them to develop a heterogeneous cell population in the tumor from which they arise [98].

Several markers have been determined so far in case of CSCs in a wide range of tumors. c-Met marker is shown to act as a potential surface biomarker in a CSC sub-population derived from different tumor sites. c-Met is a component of receptor tyrosine kinases family which plays a pivotal role in the maintenance of various important process in cellular metabolism such a cell growth, angiogenesis, metastasis, and cell survival. c-Met along-with its physiologic ligand hepatocyte growth factor (HGF) is involved in the regular cellular activities in mammalian system and also participates in the epithelial–mesenchymal communications which occur in organ morphogenesis process [99]. c-Met helps in the activation of several signaling pathways such as; NF-kB, Wnt/GSK-β-catenin, RAS-MAPK and PI3K-AKT signaling taking place inside the cell. c-Met and HGF overexpression has been reported in several carcinoma cases and stroma in the outside environment respectively. In a recent study, it was reported that in eight GCTB samples isolated from patients, c-Met + subpopulation containing stromal cells exhibit cancer stem cell like characteristics such as; self-renewability, migration capacity and differentiation potential. This subpopulation was also reported to show the ability to develop tumors in in vivo models [100].

### Role of CSC-based mTOR/PI3K-AKT signaling pathway in GCTB

The phosphoinositide-3-kinase (PI3K) signaling is categorized as one of the commonly triggered signaling pathways in several types of human cancers. This pathway is also used interchangeably as AKT pathway which helps in modulating important cellular metabolism processes such as; cell growth, proliferation, cell cycle progression and survival [101]. PI3K family including enzymes consists of three important classes which have been created on the basis of the structure and substrate specificity considered by every single enzyme. Among these three classes, Class I PI3Ks have been shown to be mostly involved in various types of cancers in human beings [102]. Akt or protein kinase B (PKB), is also considered as serine/threonine kinase and consists of three isoforms which are secluded such as; Akt1 (PKBα), Akt2 (PKBβ) and Akt3 (PKBγ). Among these three isoforms, Akt1 shows overexpression in various cancers including GCTB [103]. Autocrine/paracrine signaling generally helps in the activation of PI3K/mTOR signaling pathway in cancers. It has been confirmed that PI3K/mTOR signaling when altered/mutated, helps the normal cell to evade apoptosis and form cancer cell and helps in the generation of self-sustaining growth mechanism which leads to the development of tumors as well as cancer [104]. mTOR complex is basically a serine/threonine kinase which contributes effectively in the sequential processing of PI3K/AKT/mTOR inundation Phosphorylation process helps in the triggering of mTOR signaling [105]. It has been demonstrated that mTOR signaling pathway helps in consistency of cell growth and proliferation, as well as it highly impacts the modulation of several transcription molecules such as; S6 kinase, ribosomal protein p70, eIF4E-binding protein 1 (4E-BP1) [106]. AKT/mTOR has been confirmed as an important signaling pathway which promotes the growth of cancer stem cells(CSCs). This pathway is associated with the process in which the conversion of cancer stem cells to endothelial cells takes place. AKT/mTOR when activated helps in the early arbitration of tumor cell growth and plasticity which occurs post-surgical treatment [107]. A research study, in which a designated scrutiny was performed regarding mTOR, showed that nearly 2000 sarcomas exhibited overexpression of mTOR which confirms the critical role of AKT/mTOR pathway in GCTB patients at clinical level. Also, the correlation process in terms of mTOR overexpression was performed which was compared with increased rate of relapse with the help of Kaplan–Meier logistic regression [108]. Several researchers investigated the pivotal role of mTOR signaling in CSCs. It was observed that mTOR exhibits the regulation of expression and survival in CSCs to a greater extent [80]. mTOR being extremely sealed serine-threonine kinase observed specifically in mammals (animals and humans) consists of two important constituents, mTORC1 and mTORC2. mTORC1 consists of mTOR, regulatory-associated protein of mTOR (Raptor), mLST8 protein and PRAS40 (proline-rich AKT substrate 40 kDa). mTORC2 includes mTOR, rapamycin-insensitive companion of mTOR (Rictor), mLST8 and mammalian stress-activated protein kinase interacting protein (mSIN1). mTORC1 forms a nutrition- and rapamycin-sensitive composite, whereas mTORC2 does not react to rapamycin. mTORC1 plays satisfactory part in case of controlling homeostasis in tissues along-with the important other processes such as; leukemogenesis, hematopoietic purpose and cell proliferation etc. mTORC2 is a key regulator of several cellular functions such as; actin remodeling, cell-cycle progression, and cell survival. mTORC2 activates AKT by the process of phosphorylation and stimulates the changes in cell survival and cell metabolism. mTOR signaling pattern helps the cancer cells and CSCs to maintain stem cell characteristics like; self-renewal, survival and cell proliferation [109]. But, the mechanism involved in the expression of CSCs through mTOR signaling is still not understood completely. Recently, mTOR signaling was investigated in human primary nasopharyngeal carcinoma (NPC) and it was found that mTOR involvement in NPC shows high impact on the behavior of CSCs thus playing a potential role in the regulation of CSC environment. Rapamycin has been used to inhibit the CSCs by specifically targeting mTOR signaling pathway which is an important innovation done so far. It helps in the improvement of therapeutic tools being used to treat several cancers such as; GCTB, NPC. and so on [110].

**Fig. 4:**
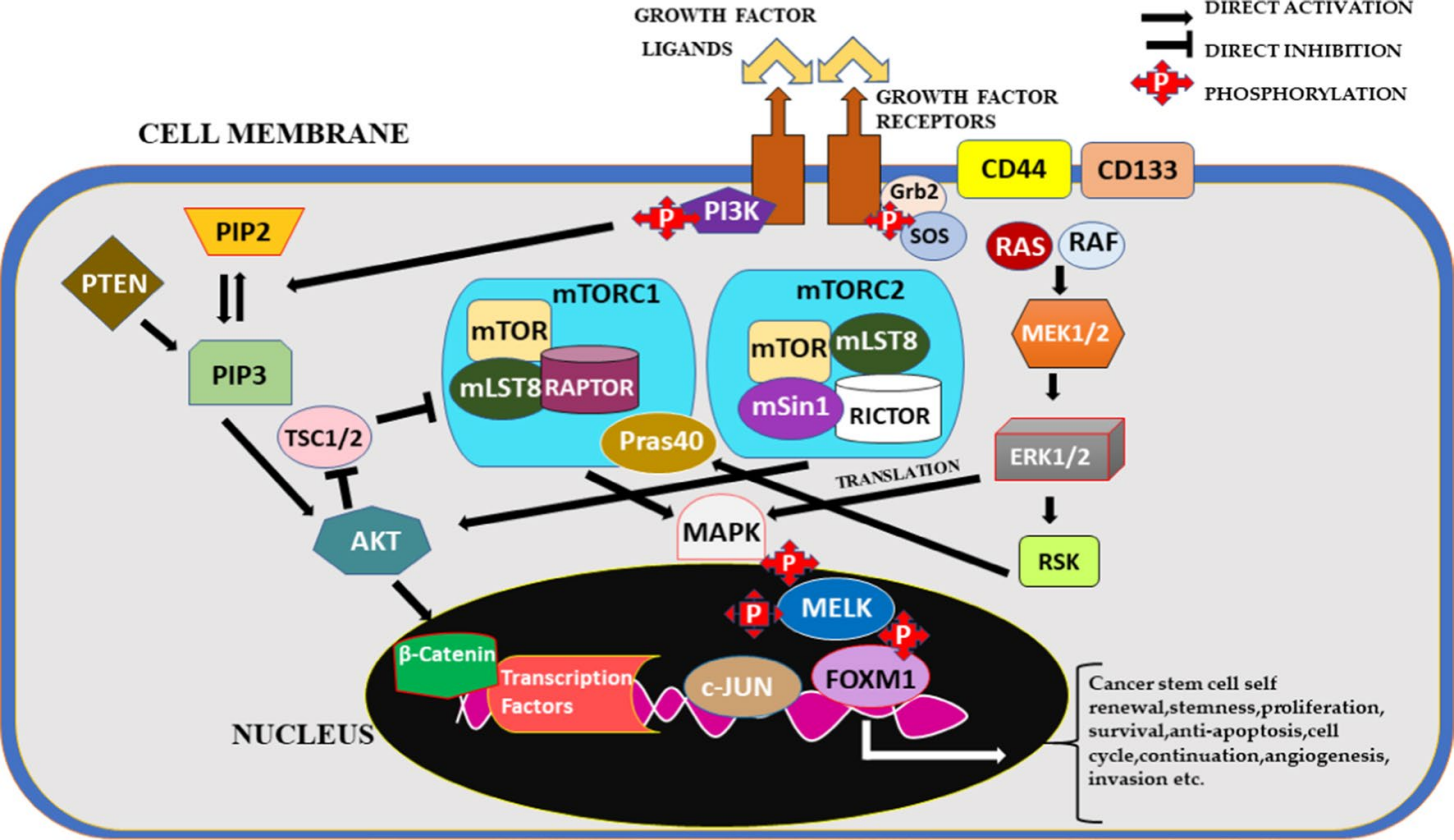
mTOR signaling through AKT and mTORC complex in combination with the downstream molecules leading to the development of cancer stem cells with several characteristics such as; self-renewal, survival, stemness, proliferation, invasion etc

mTOR signaling pathway is basically helped by receptor tyrosine kinase (RTK) to continue its function in upstream pattern. RTK activates phosphoinositide 3-kinase (PI3K), which leads to the phosphorylation process of several molecules acting as downstream effectors. p110 gets inhibited by p85 which actually facilitates this inhibition in the plasma membrane where class I PI3Ks get engaged and help p110 to phosphorylate phosphatidylinositides 4,5-biphosphate (PIP2) and develop phosphatidylinositides 3,4,5-triphosphate (PIP3). PIP3 is a lipid product which contributes effectively as a second messenger in mTOR/AKT pathway. PIP3 helps in the triggering of AKT-dependent and AKT-independent downstream signaling pathways as observed by Vanhaesebroeck et al. in 2010 [111]. PH domain in the N-terminal region of the kinase recruits PIP3 in the plasma membrane for AKT activation after translocation. AKT signaling occurs in a follow-up process of mTOR complex 1 and 2 post-activation in a downstream fashion [112]. mTOR signaling pathway constitutes mTOR as a potential downstream signal activated in giant cell tumor of bone(GCTB). The assembly of mTOR is composed of FAT domain, N-terminal tandem HEAT repeats, C-terminal kinase domain, FATC domain and FRB domain. The two discrete mTOR complexes labelled as mTOR complex 1 (mTORC1) and mTOR complex 2 (mTORC2) as shown in Fig. 4 contribute actively to the entire signaling mechanism in CSCs involved in GCTB [113]. mTOR complex 1 has the ability to undergo auto-phosphorylation, thereby activating several downstream proteins such as; p70S6 kinase (p70S6K), which in turn contributes to the acceleration and maintenance of the cell cycle [114]. The phosphorylation of 4EBP1 results in the release of eIF4E. Consequently, the free eIF4E enriches the translation of c-myc, cyclin D1, VEGF, and matrix metalloproteinase-9 (MMP-9), thus stimulating the cell survival, angiogenesis, invasion and metastasis processes [115]. AKT phosphorylates tuberous sclerosis complex 2(TSC2) thereby activating mTOR and restricting the effect of TSC2 over mTOR complex 1. The tuberous sclerosis complex (TSC) is composed of two complexes, Tuberous sclerosis complex 1 (TSC1; Hamartin) and Tuberous sclerosis complex 2(TSC2; Tuberin). TSC acts as an important negative regulatory unit of mTORC1 function.TSC1 prevents TSC2 from degradation and plays an important role as a stabilizer for TSC2 [116]. The phosphatase and tensin homolog (PTEN) functions as a basic negative regulatory unit of AKT/mTOR-PI3K pathway. PTEN erases 3-phosphate from PIP3 which makes PIP3 lose a phosphate group and reduce to PIP2 as observed by Chia et al. in 2010 [117, 118]. When Akt gets phosphorylated, several downstream targets like; mTOR get triggered to help in the continuity of cell metabolism. mTOR is an important downstream signal of PI3K/AKT/mTOR signaling pathway activated in head and neck cancer [119]. A series of factors regulate mTORC1 including; nutrients, oxygen and cellular stress, growth factors and energy status. mTORC1 helps in the encouragement of proliferation, cell survival, ribosome biogenesis, angiogenesis, protein synthesis, migration, invasion, and metastasis by phosphorylating MAPK which in turn triggers MELK inside the nucleus through phosphorylation and releases c-JUN, FOXM1 via. translation process of several proteins [120, 121]. mTORC2 is a key regulator of several cellular functions such as; actin remodeling, cell-cycle progression, and cell survival. This activation also leads to the blockade of various pro-apoptotic proteins such as; p53 and Bcl-2 associated death promotor (Bad) [122]. mTORC2 functions in actin remodeling, cell-cycle progression, and cell survival mTOR complex 1 (mTORC1) helps in the triggering of mTOR, which activates several proteins like; cyclin D, these activated proteins help in the promotion of cell cycle, the pro-apoptotic proteins including eukaryotic translation elongation factor 2 kinase (eEF-2 K) and cyclin-dependent kinase inhibitors including p27kip1 are also suppressed [123]. mTORC2 has been shown to play an important role in case of cytoskeletal modulation, cellular metabolism as well as Akt activation processes. The role AKT/mTOR signaling pathway plays downstream of the epidermal growth factor receptor (EGFR) was investigated by Gursel et al. in mouse-derived astrocytic primary cell lines, which exhibit much resemblance with glioblastoma. Few studies also confirmed that Akt and mTOR play a vital role in order to exhibit the reliability of CSCs in several cancers such as; GCTB, neuroblastoma and glioblastoma [124, 125].

Various studies worked on effective targeting of mTOR pathway in order to inhibit the CSCs because these cells develop chemo resistance against conventional radiotherapy as well as chemotherapeutics. In several cancer cells such as; gastrointestinal cancer cells and liver tumor cells, it was shown that mTOR when suppressed, helps in the proliferation of CSCs. It was investigated by Bleau et al. that upon inhibition of Akt, the modulation of the function of ATP binding cassette transporters (ABCG2) occurs which in turn leads to the development of the chemo resistance in glioma tumor stem-like cells [126].

### Role of mesenchymal stem cells (MSCs) in GCTB

Mesenchymal stem cells (MSCs) are considered as a type of stem cells which are multi-potent in nature and present in a variety of adult tissues. MSC is a single cell which has the ability to develop into cartilage, bone and several other mesenchymal tissues [127]. The derivation of GCTSCs from MSCs has been confirmed by the expression of several MSC exemplary markers associated with the cell surface. The significant potential of MSCs to differentiate into osteoblasts, adipocytes as well as chondrocytes also adds to the confirmation of GCTSC origin [128]. It was confirmed from recent studies that the mineralized nodules get developed in GCTSCs osteogenically under in vitro environment due to deposition of mineralization matrix and increased alkaline phosphatase activity [129]. This was confirmed with the help of von Kossa staining and increasing alkaline phosphatase enzyme activity which was scheduled for 4 weeks of stimulation as per the reports. GCTSCs undergo chondrogenic intruding which results in the expression of collagen type II observed in stimulated cells. In case of adipogenic stimulation, there was an exhibition of accumulation of lipid droplets observed in GCTSCs in the cytoplasm with the help of Sudan red staining [130]. According to recent studies, the development of GCTSCs observed due to the expression of several MSC markers has helped as pivotal evidence in case of GCTs to possess MSC origin. Several control experiments carried out under in vitro conditions with the help of double staining in which the proliferation marker anti-MDM2 and either SH2 or anti-CD166 were used, confirmed the origin of GCTs as MSCs. GCTSCs being important part of tumor cells of the GCT has also been confirmed by non-substantial increase in the progress of GCTSC proliferation [131]. Analysis of CD166 and CD105 mRNA were also analyzed in which it was found that the expression in the early MSC markers is responsible for the development of GCTSCs. Mesenchymal stem cell markers such as; CD166, CD73, CD105 have been shown to get expressed via. sub-population of stromal cells. Also, the expression of mesenchymal markers FGFR3 (fibroblast growth factor receptor (3), CD34, collagen type-IIa has been witnessed in recent research studies. Neoplastic GCTSCs generation from MSCs has obtained a potential support from the previous research which confirms this link with strong evidence [132]. The recent findings confirmed that CSCs could play an important role in the development of GCT which could be derived from monocytic/osteoclastic or mesenchymal/osteoblastic cells. The existence of pluripotent stem cells in GCT has also been confirmed recently, these cells have the ability to differentiate into hematopoietic as well as mesenchymal cell types which have been shown to play a prominent role into the development of GCT [86]. With the help of recent reports, it has been confirmed that interleukin-6 helps in the development of a mesenchymal phenotype, whereas the stem cell factor if added, will be very much helpful to develop a hematopoietic phenotype of the pluripotent stem cells. It was also shown that interleukin-6 showed a significant increase in case of expression in GCTSCs, which may have the chance to lead to the formation of mesenchymal phenotype in GCTSCs [133].

The advancement of the research studies in case of finding Stro1 antigenic determinant in GCTSCs emerged as a ray of hope to study the progression of GCTSCs as well as the relation with osteoblastic lineage in a wide range of tumors. The researchers observed this determinant as the depiction of the antigen which was determined by the Stro1 antibody acting as a specific marker for osteoprogenitor cells prior to the cells expressing alkaline phosphatase (ALP) [134]. It was observed that in majority of human fibroblast colony-forming units which are detective, particularly the undifferentiated osteoblastic precursors and approximately 85% of bone marrow stromal cells, Thy1.1 expressed at a reasonable rate [135]. According to the recent research studies, successful beginning of the differentiation into osteoblasts, chondroblasts, and adipocytes, were matched with the results Joyner et al. reported in 1992 in which GCTSCs were targeted in order to express ALP and develop mineralized nodules under in vitro conditions and the findings were found to be uniform. GCTSCs were also targeted by some researchers to generate bone when injected subcutaneously into SCID mice under in vivo environment [136]. It was shown few decades ago that the monocytic/osteoclastic cells could be responsible to develop GCTB. These pluripotent stem cells have been shown to be present in mice and humans to a greater extent. With the help of some specific potential antibodies, some effective cell populations have been recognized which help in the testing process in order to confirm the presence of such pluripotent stem cells in GCT. With the help of characterization of pluripotent stem cells in GCTB, prominent link has been established in case of the role of stem cells in the development of GCTB [137]. GCTSCs have shown to express several osteoblastic markers, osteonectin, osteocalcin, osteopontin etc., as well as these cells undergo osteoblastic differentiation that has been confirmed recently under both in vivo as well as in vitro conditions thus acting as a potential link relating these cells to the osteoblastic lineage [138].

### Role of CSC-based microRNAs in GCTB

MicroRNAs (miRNAs) associated with stem cells have been found to exhibit a potential contribution in the development of a wide range of tumors. MicroRNAs being small non-coding, single-stranded RNA molecules with the help of introducing mRNA degradation or inhibition of translation regulate their target genes in a negative manner [139]. In case of the regulation of a majority of biological functions such as; cell growth, differentiation and apoptosis, miRNAs have been confirmed to contribute significantly in these biological processes. Recent reports show that cancer stem cells develop several types of tumors with the help of insubstantial locations in the chromosomes where gene coding for miRNAs occurs [140]. Stem cell outcome as well as its behavior gets regulated by miRNAs to a greater extent. There is a potential evidence regarding the stem cell-based miRNAs showing that these miRNAs help in the regulation of various important cellular functions including; self-renewal characteristics, cell differentiation as well as cell division [141]. miRNAs control various processes in cancer stem cells which help in the development of cancer. These stem cell-based microRNAs have been confirmed by researchers recently to be very much helpful in finding the important molecular mechanisms through which neoplastic transformation of MSCs take place and which are highly responsible for the development of GCTs [64]. The apoptosis inhibitor API5 in transcriptional regulation via. miR-224 as well as the identification of three novel mir-224 target genes and their overexpression in GCTSCs when compared to MSCs were recently confirmed with the help of research studies. These genes are involved into some core cellular processes such as; apoptosis resistance, proliferation, differentiation and regulatory mechanism of gene transcription etc. which shows that these genes have an adequate importance in the contribution towards the neoplastic transformation of MSCs [142]. In order to study the role of miRNAs in the process of neoplastic transformation of MSCs which leads to the development of GCT, several miRNA expression patterns consisting of GCTSCs and MSCs were clearly analyzed by researchers with the help of which differentially expressed candidate miRNAs and miRNA target genes were determined. These target genes could be helpful for the development of new approaches and strategies to effectively treat these types of cancers [143].

The screening process was performed by researchers for microRNA impression differentiating GCTSCs from MSCs in case of determination of certain important factors which were non-intervened and which take part in the initiation step of tumorigenesis in GCTB. The expression of eight suitable molecules of microRNAs in GCTSC was re-established and functional in vitro analysis module was taken into account to analyze the influence of this expression on the neoplastic phenotype of these cells [144]. The miRNAs such as; mir-127-3p and mir-376a-3p when expressed repeatedly, helped in the determination of potential and continuous impact on various important cellular processes like proliferation, migration, formation of spheres, formation of colonies etc. The fixed Dlk1- Dio3 locus present on chromosome 14 is subjected to abatement through epigenetic mechanism in GCTB in which these miRNAs have been confirmed to be present in a conglomeration manner [145]. In several cancers such as breast cancer, miR-127-3p was shown to down-regulate in which it was also determined that low miR-127-3p expression leads to metastasis in lymph node as well as clinical stage was shown to be highly modified. In addition, epigenetic alterations help in the development of mutations in the tumor cells in GCTB which take place specifically in miR-127-3p led by H3F3A mutations [146]. Recently, various specific targets such as; COA1, SLC25A23, SLC25A1 and FAM57A were determined by researchers with the help of microarray and RT-qPCR techniques through which expression profiling of miR-127-3p was performed in transfected cells. According to the reports, it was shown that when miR-127-3p was re-established, it mostly targeted the expression of SLC25A1 and COA1 which play an important role in energy metabolism process by encoding the proteins present in mitochondria. Apart from miR-127-3p, it was shown that miR-376a-3p when replaced, exhibited a significant influence in case of phenotype which is known to be neoplastic developed in GCTSCs [147]. MiR-376a-3p undergoes down-regulation in colon cancer melanoma, prostate cancer and hepatocellular carcinoma as per the reports. In recent studies, it was found that the replacement of miR-376a-3p helps in the reduction of colony formation, proliferation, migration, and the sphere formation characteristics in the GCTSCs [148]. Internal miR-376a-3p amount when increased in a cell leads to the considerable suppression in the GLE1, PDIA6, PON2 and TFAM gene expressions. Luciferase reporter assay performed for GLE1 and PDIA6 helps in the determination of explicit communication of miR-376a-3p with such contemporary target genes. It has been shown that GLE1 helps in the management of activation of translation as well as contributes significantly towards the modulation of gene expression with the help of regulating the transfer of mRNA from the nucleus [149]. In this way, it was recently observed that miR-127-3p and miR-376a-3p when silenced in GCTSCs plays a vital role in the process of alimentation of these cells associated with neoplastic phenotype. With the help of recent studies, eight possible new genes were determined which act as target genes. This finding added to the enunciation of the influence related to the re-establishment of microRNA for COA1, GLE1 and PDIA6 along-with miR-127-3p and miR-376a-3p which help in the blockade of tumor development by playing a potential role as direct target genes [37].

## Conclusion and perspectives

This review talks about the giant cell tumor of bone in general and how cancer stem cells play a vital role in the development of GCTB which is highly important in research aspects nowadays. Bone tumor is developed when a normal bone cell evades apoptosis and leads to the uncontrolled growth and generates a huge mass of cells which is called as bone tumor. The multi-nucleated giant cells resemble to osteoclasts to a greater extent biochemically, these cells have fixed nuclei which in turn resemble to the nuclei existing in stromal cells. These types of giant cells are commonly seen in a large number of primary bone tumors. There is a great concern nowadays about the stem cell sub-population playing a prominent role in the development of GCT. This issue needs to be understood clearly and addressed in a proper manner through advanced research and highly effective techniques. Much more knowledge is required to understand the in vivo mechanism of development of PGCCs activated by CoCl_2_ and the PGCCs developed under the impact of physiologic conditions of hypoxia. Besides, the development of chemo resistance in PGCCs is to be clearly understood in order to increase the usage of effective chemotherapeutics in correct ways. Besides, c-Met, mTOR/PI3K-AKT signaling mechanism etc play a significant role in developing CSCs and activating various downstream activities in a wide range of human cancers including GCTB. CSC associated miR-127-3p, miR-376a-3p and other miRNAs along with their target genes might be the potential points to focus upon in future to work towards developing novel therapeutic strategies which could be highly effective against neoplastic stromal cell population generated within GCTB. The process of silencing of miR-127-3p and miR-376a-3p., etc being studied in various types of tumors highlights the promising importance of newly recognized target genes in case of treating GCTB as well as other tumor sources effectively. There is a need of potential understanding in case of the molecular mechanisms playing an important role in the process of transformation and also the factors which interfere positively and are expressed as potential targets in developing highly advanced and effective therapies.

## Data Availability

Not applicable

## Abbreviations

GCT: Giant cell tumor
GCTSCs: Giant cell tumor stromal cells
GCTB: Giant cell tumor of bone
PGCCs: Polyploid giant cancer cells
CSCs: Cancer stem cells
MSCs: Mesenchymal stem cells
miRNAs: Micro-ribo nucleic acids
